# Multiple left ventricular thrombi in a patient with dilated cardiomyopathy and cerebral infarction: a case report

**DOI:** 10.1186/1752-1947-8-306

**Published:** 2014-09-13

**Authors:** Takanao Mine, Ikuo Sato, Hiroji Miyake

**Affiliations:** 1 Department of Internal Medicine, Cardiovascular Division, Hyogo College of Medicine, 1-1 Mukogawa-cho, Nishinomiya 663-8501, Japan; 2 Department of Clinical Laboratory, Nishinomiya Kyoritsu Neurosurgical Hospital, 11-1 Imazu-yamanaka-cyo, Nishinomiya 663-8211, Japan; 3 Department of Neurosurgery, Nishinomiya Kyoritsu Neurosurgical Hospital, 11-1 Imazu-yamanaka-cyo, Nishinomiya 663-8211, Japan

**Keywords:** Left ventricular thrombi, Anticoagulation therapy, Dilated cardiomyopathy, Cerebral infarction

## Abstract

**Introduction:**

It is not clear whether patients with sinus rhythm and reduced left ventricular function should be treated with anticoagulation therapy during or after treatment for heart failure.

**Case presentation:**

A 67-year-old Japanese man was hospitalized at our institution with heart failure due to dilated cardiomyopathy. On day after discharge, he developed cerebral infarction and showed persistence of multiple left ventricular thrombi. His paralysis completely improved at 2 days after edaravone and heparin administration; however, his left visual field defect persisted.

**Conclusion:**

Patients in sinus rhythm with reduced left ventricular function might benefit from anticoagulation therapy during treatment for heart failure.

## Introduction

We occasionally encounter patients who develop cerebral infarction during treatment for heart failure. Embolic events are estimated to occur in 4% of patients with dilated cardiomyopathy who have a left ventricular ejection fraction (LVEF) ≤35% [1]. Further, the incidence of LV thrombus in patients with dilated cardiomyopathy and sinus rhythm is 13%, and the clot is located in the left atrial appendage in 68% of these cases [2]. Although guidelines state [3] that patients with atrial fibrillation should be treated with anticoagulation therapy, the benefits of warfarin for patients with heart failure in sinus rhythm have yet to be established [4]. Indeed, the reduced risk of ischemic stroke in response to warfarin is offset by a risk of hemorrhage in patients with reduced LVEF [5]. Therefore, it is not clear whether patients with sinus rhythm and reduced LV function should be treated with anticoagulation therapy during or after treatment for heart failure. In this report, we describe a case of a patient with hypertensive cardiomyopathy and in sinus rhythm who experienced left cerebral infarction related to LV thrombi after treatment for heart failure.

## Case presentation

A 67-year-old Japanese man was hospitalized at our institution with heart failure due to dilated cardiomyopathy. He was not taking any medications and had a history of mild diabetes mellitus. He was discharged after 1 week of treatment with vasodilators and diuretics. Electrocardiographic monitoring during his hospitalization showed sinus rhythm.

**Figure 1:**
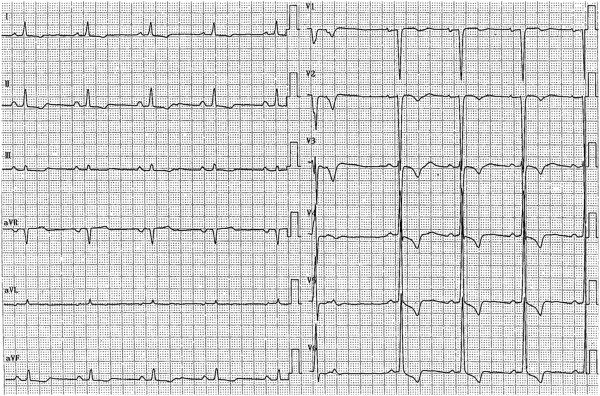
Electrocardiogram obtained upon admission.

**Figure 2:**
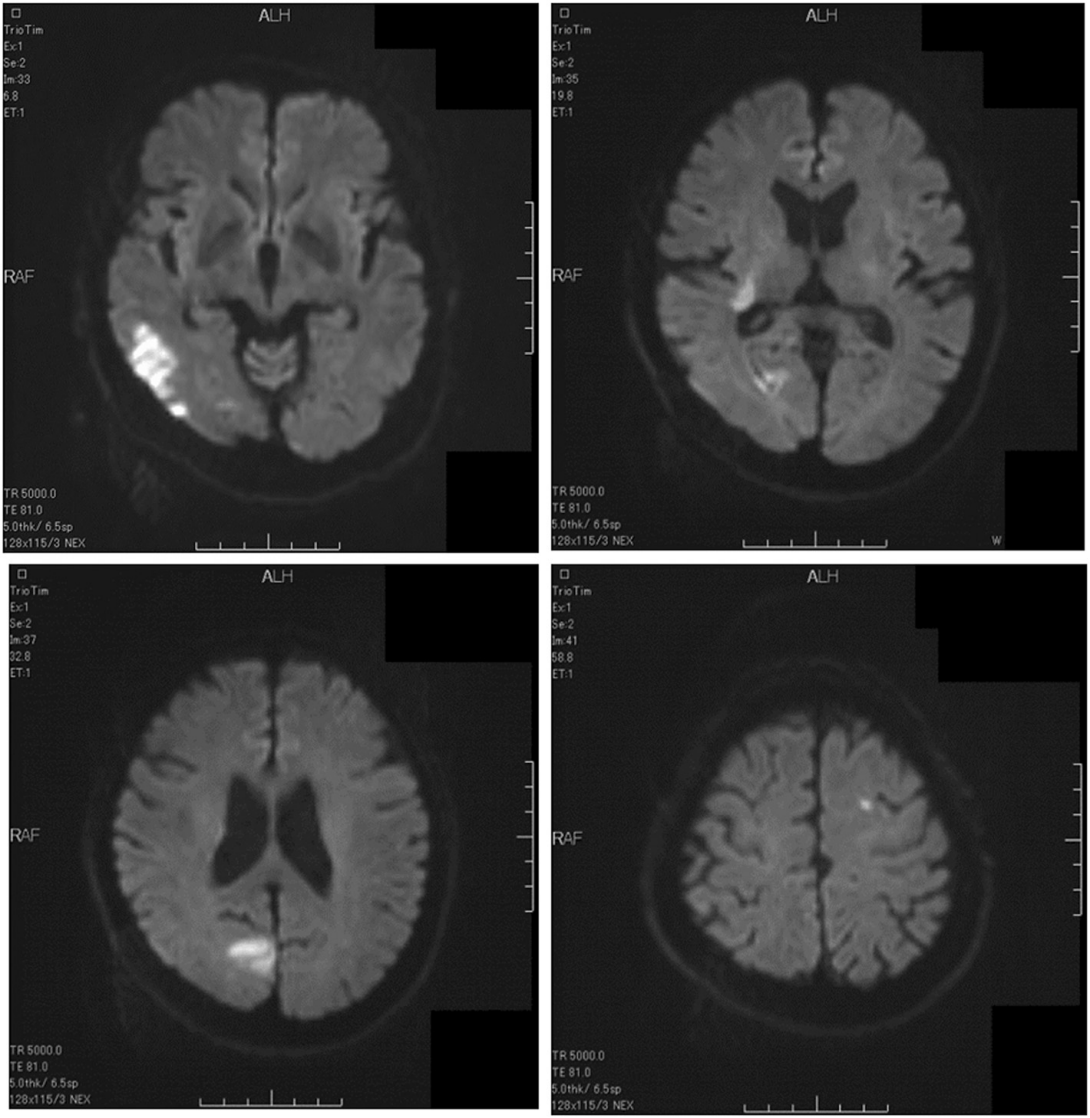
Brain magnetic resonance imaging scans show multiple cerebral infarctions.

**Figure 3:**
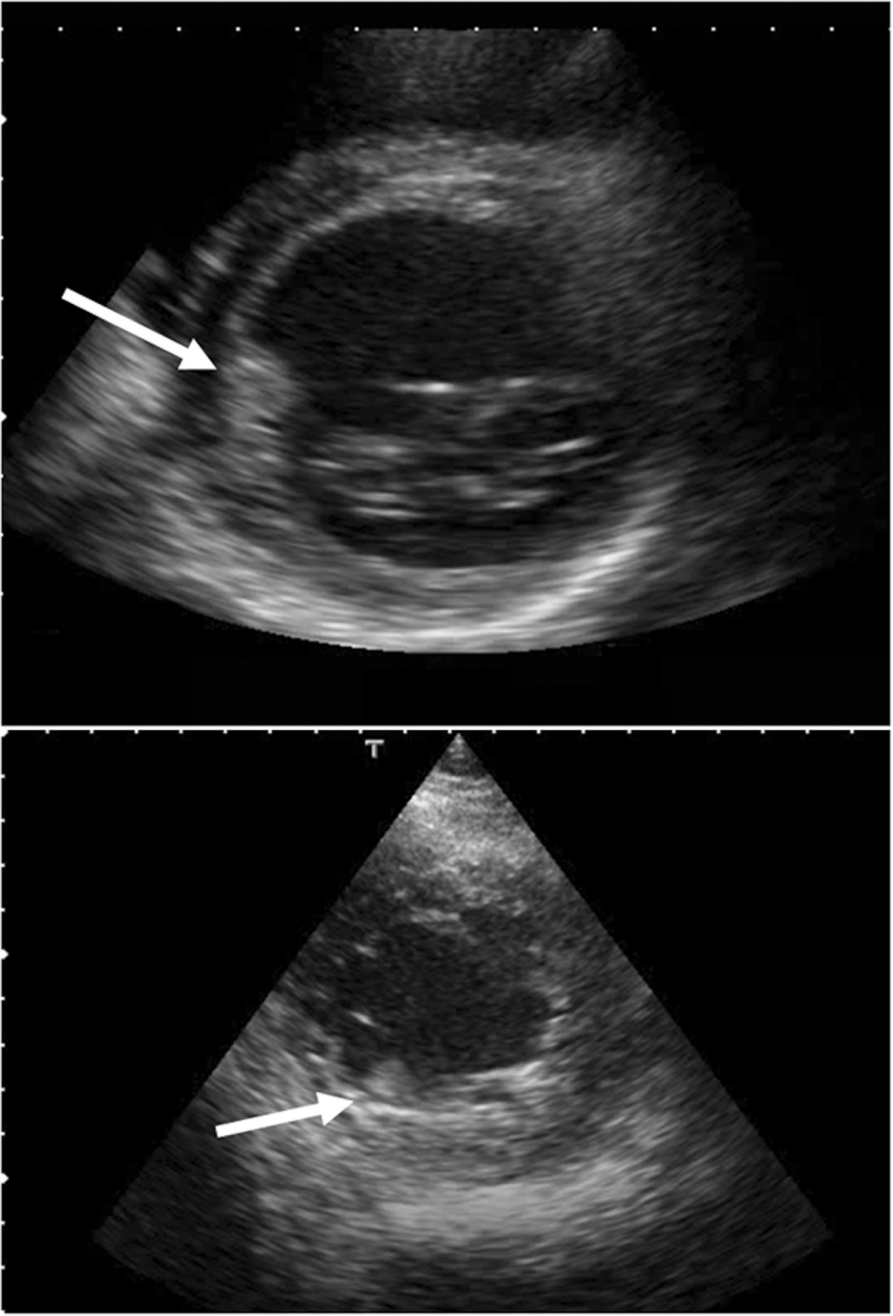
**Short-axis view echocardiograms.** Arrows point to two mobile thrombi.

On the day after discharge, the patient presented to our emergency unit with left-limb paresis and left visual field defect. His medications at that time consisted of bisoprolol, enalapril, digoxin and metformin. A physical examination showed that his blood pressure was 119/67mmHg, heart rate was 60 beats/min and his respiratory rate was 10 breaths/min. He was alert, and an electrocardiogram obtained upon admission showed LV hypertrophy, premature ventricular contractions and sinus rhythm with a heart rate of 57 beats/min (Figure 1). A chest X-ray showed cardiomegaly, and laboratory testing indicated high plasma levels of D-dimer (3.9μg/L) and brain natriuretic peptide (834pg/ml) and elevated transaminases (aspartate aminotransferase, 38IU/L; alanine aminotransferase, 64IU/L). Brain magnetic resonance imaging showed multiple cerebral infarctions in the occipital lobe, corona radiata and left frontal lobe (Figure 2). Echocardiograms indicated LV dysfunction, a LVEF of 27%, a LV end-diastolic diameter of 66mm and two mobile thrombi at the basal septum and apical inferior portions of the left ventricle (Figure 3, Additional file 1).

His paralysis improved at 2 days after edaravone and heparin administration; however, his left visual field defect persisted. After 6 days of heparin therapy, the patient was initiated on warfarin, which led to disappearance of the two mobile left ventricle clots. No evidence of embolism was seen during treatment. We conclude that this patient had multiple cerebral infarctions due to LV thrombi immediately after treatment for heart failure. These observations highlight that LV thrombi can occur in patients in sinus rhythm who have reduced LV function and are undergoing treatment for heart failure. Fortunately for our patient, his thrombi disappeared after initiation of anticoagulation therapy.

## Discussion

There are several reports of multiple LV thrombi in patients with LV noncompaction [6], arrhythmogenic right ventricular cardiomyopathy [7] and dilated cardiomyopathy [8,9]. However, persistence of multiple thrombi in the left ventricle in a patient with dilated cardiomyopathy after stroke is extremely rare.

Anticoagulant therapy (for example, warfarin, heparin, dabigatran) is used for the treatment of intracardiac thrombi [10]; however, the use of anticoagulant therapy for patients with reduced LV function and in sinus rhythm remains controversial. The benefits of warfarin for patients in sinus rhythm have not been established for those with heart failure [4]. No significant overall difference was observed in mortality, intracereberal hemorrhage or ischemic stroke when the use of warfarin versus aspirin was compared in patients with reduced LVEF [5]. It is not clear whether anticoagulant therapy is needed in patients with reduced LV function and heart failure. In one study, elderly patients presenting with acute ischemic stroke were found to have high plasma osmolality levels and showed dehydration [11]. Dehydration after acute ischemic stroke is strongly independently associated with venous thromboembolism [12]. In our patient, LV thrombi formed during treatment for heart failure and resulted in cerebral infarction. Because coagulability may be enhanced by the administration of diuretics, the use of anticoagulants during treatment of our patient for heart failure might have prevented his cerebral infarction. Therefore, we reason that patients in sinus rhythm and with reduced LV function might benefit from anticoagulation therapy during treatment for heart failure. However, there are no guidelines which recommend the use of anticoagulants in heart failure during treatment. It is necessary to study the incidence of cerebral infarction and evaluate dehydration during treatment in patients with heart failure.

## Conclusion

In this report, we describe a case of a patient with dilated cardiomyopathy who had persistent multiple LV thrombi after experiencing a stroke. This finding suggests patients in sinus rhythm with reduced LV function might benefit from anticoagulation therapy during treatment for heart failure, but review of additional cases is needed to validate this conclusion.

## Consent

Written informed consent was obtained from the patient for publication of this case report and any accompanying images. A copy of the written consent is available for review by the Editor-in-Chief of this journal.

## Abbreviations

LVEF: Left ventricular ejection fraction.

## Competing interests

The authors declare that they have no competing interests.

## Authors’ contributions

TM drafted the manuscript and contributed important intellectual content. TM, IS and HM made substantial editorial revisions to the manuscript. HM made major contributions to the conception and design of the report. All authors read and approved the final manuscript.

## Supplementary Material

Additional file 1 Video of echocardiogram shows two mobile thrombi in the left ventricle. Click here for file
